# ASSOCIATION BETWEEN FUNCTIONAL LIMITATIONS AND SOCIAL ISOLATION AMONG PEOPLE WITH AND WITHOUT DEPRESSIVE SYMPTOMS

**DOI:** 10.1093/geroni/igae098.3427

**Published:** 2024-12-31

**Authors:** Chuwen Zhong, Briana Mezuk, Linh Dang

**Affiliations:** University of Michigan, Ann Arbor, Michigan, United States; University of Michigan, Ann Arbor, Michigan, United States; University of Michigan, Ann Arbor, Michigan, United States

## Abstract

Functional limitations are prevalent among older adults with depression, and prior research suggests that social isolation can be an underlying mechanism. This study examines whether the structure of linkages between functional limitations and social isolation differs by the presence of cardinal depressive symptoms (dysphoria/anhedonia) using network analysis. Data came from the 2014/2016 Health and Retirement Study (n=7,270). Cardinal depressive symptoms were assessed using the Composite International Diagnostic Interview. Isolation (n=14 nodes) and functioning (n=16 nodes) were measured using multiple indicators (e.g., having a partner, balance test, feeling isolated, having difficulty dressing). We compared the centrality, network density, network structure, global strength, and correlation of edges between the networks of respondents who did vs. did not have cardinal symptoms of depression. Respondents who had depressive symptoms (n=628, 8.64%) were younger and more likely to be female. Compared to those without depressive symptoms, the network of those with depressive symptoms had a lower density of edges (ratio = 0.14 vs 0.20) and had different central nodes (lack of common peers vs difficulty taking medications, respectively). However, the network comparison tests showed that the two networks were similar in terms of correlation of edges (Pearson r = 0.81,p< 0.01), network structure (Network Invariance Test statistic = 1.82, p = 0.48), and strength (Global Strength Invariance Test statistics = 35.28, p = 0.79). Findings suggest that the network linkages between functional limitations and social isolation do not substantially differ as a function of cardinal depressive symptoms.

